# East and West in the Medical School

**Published:** 1918-03-16

**Authors:** 


### EAST AND WEST IN THE MEDICAL SCHOOL.

In no direction has the lead of Western scientific investigation been more enthusiastically followed by students from the Far East than in the domain of medicine and surgery. Medical schools receive a constantly increasing influx of eager and promising students from the Indian Empire, and foundations are being laid on which in the end India may rear its own structure of medical education and may profoundly influence, it may well be believed, the future of pathological research. For the present, however, degrees have to be won in this country, and the fact that in British schools practice goes hand-in-hand with theory is responsible for creating problems which ought not to be ignored. Side by side with all class distinctions in this country there exists a deep unexpressed sense of equality, linking men together by a thousand common ties. In the East this sense of equality is less, if not, under many conditions, altogether wanting. The quality of individuality is richly developed in these islands, and the inmates of hospitals and infirmaries who form the doctor's clinical material are all "characters" requiring in those who minister to their needs some degree of perception, obedience to a certain code of etiquette, and, above all, some evidence of brotherly kindness. In the vast majority of men and women medical students the right manner towards the patient is inbred—a product of birth, home influence, education, and social environment. Some may be, to tell the truth, off-hand, and apparently insensible to the suffering they witness. Yet they handle successfully men and women who understand their awkward reserve, and are quick to recognise the spirit of humanity stirring under a disguise which is perfectly familiar to them.

Has it been sufficiently taken into account that medical students from other parts of the Empire have to learn what comes naturally to the British-born—the art of dealing successfully with British patients? It is asking for trouble to put men and women of a different race into positions of authority in British institutions, or, indeed, to perform any duties in them without helping them first to acquire the code. Many patients are full of narrow prejudices; all are primed with a preconceived idea of the manner in which they ought to be addressed. It is fatally easy for those who stand outside their lives and interests to wound their feelings, irritate and alarm them. Such blunders do not make for recovery or efficiency, to say the least of it. At the same time, a certain halo of distinction hangs over any "foreigner" who takes pains to make his presence acceptable, and some are extraordinarily adaptable to European methods. There is perhaps no better mode of inculcating the right way with patients than through such courses of nursing demonstrations as used to be delivered to students in pre-war days at the London and, if we remember rightly, at St. Bartholomew's Hospital. The matron or sister is the best observer of things likely to arouse antagonism in the patient, and most of the little acts of commission and omission on the part of students from other countries, which are at present a stumbling-block, could be obviated by the kind of instruction we have indicated. Moreover, such courses would add very greatly to the value of practitioners when returning to positions of responsibility in their own country. The art of nursing, as it is taught in the best of our training-schools, is the art of humanity towards the sick. It is this art which all members of the Empire must learn, if their scientific studies are not to lead them into a barren and dry land, where no water is.

